# SGLT2 inhibition, venous thrombolism, and death due to cardiac causes: a mediation Mendelian randomization study

**DOI:** 10.3389/fcvm.2024.1339094

**Published:** 2024-05-13

**Authors:** Lili Shi, Xiupan Wei, Jinlan Luo, Ling Tu

**Affiliations:** ^1^ Department of Geriatric Medicine, Tongji Hospital, Tongji Medical College, Huazhong University of Science and Technology, Wuhan, China; ^2^ Hubei Key Laboratory of Genetics and Molecular Mechanisms of Cardiological Disorders, Wuhan, China; ^3^ Department of Rehabilitation Medicine, Zhongda Hospital, Southeast University, Nanjing, China

**Keywords:** SGLT2 inhibition, venous thrombolism, mortality rates, cardiac events, Mendelian randomization

## Abstract

**Objective:**

To investigate the causal role of venous thrombolism mediating sodium-glucose cotransporter 2 (SGLT2) inhibition in death due to cardiac causes using Mendelian randomization (MR).

**Methods:**

A two-sample two-step MR was used to determine (1) the causal effects of SGLT2 inhibition on death due to cardiac causes; (2) the causal effects of venous thrombolism on death due to cardiac causes; and (3) the mediation effects of venous thrombolism. Genetic proxies for SGLT2 inhibition were identified as variants in the SLC5A2 gene that were associated with both levels of gene expression and hemoglobin A1c. Additionally, employing MR to investigate the causal association between SGLT2 inhibition and cardiac arrest as well as coronary heart disease (CHD).

**Results:**

SGLT2 inhibition was associated with a lower risk of death due to cardiac causes (odds ratio [OR] = 0.983, [95% CI = 0.972, 0.993], *P* = 0.0016). Venous thrombolism was associated with death due to cardiac causes ([OR] = 1.031, [95% CI = 1.005, 1.057], *P* = 0.0199). Mediation analysis showed evidence of indirect effect of SGLT2 inhibition on death due to cardiac causes through venous thrombolism [β = −0.0015, (95% CI = −0.0032 −0.0002), *P* = 0.042], with a mediated proportion of 8.9% (95% CI = 1.2%, 18.7%) of the total. Furthermore, SGLT2 inhibition was linked to a lower risk of cardiac arrest ([OR] = 0.097, [95% CI = 0.013, 0.742], *P* = 0.025). SGLT2 inhibition was linked to a lower risk of CHD ([OR] = 0.957, [95% CI = 0.932, 0.982], *P* = 0.0009).

**Conclusions:**

Our study identified the causal roles of SGLT2 inhibition in venous thrombolism. SGLT2 inhibition may influence death due to cardiac causes through venous thrombolism. Additionally, SGLT2 inhibition was associated with reduced risk of cardiac arrest and CHD.

## Introduction

Sodium-glucose cotransporter 2 (SGLT2) inhibitors, such as dapagliflozin, and canagliflozin, are antidiabetic medications that function by impeding the glucose reuptake in the renal tubule, as well, as facilitating the secretion of glucose through the urinary system (1). Extensive research unequivocally has confirmed the substantial therapeutic effects of SGLT2 inhibitors in mitigating cardiovascular disorders, addressing heart failure, and enhancing renal function (2). Furthermore, growing findings indicated the potentiality of reducing the hazard of cardiac-related death, but a causal relationship between SGLT2 inhibition and cardiac-related death has not been reported. Cardiac arrest and coronary heart disease (CHD) stand as the predominant contributors to mortality due to cardiovascular diseases. The causal link between SGLT2 inhibition and both cardiac arrest and CHD remains uncertain.

Venous thrombolism is a common complication of cardiac events including heart failure (3), atrial fibrillation (4), and myocardial infarction (5). A study conducted a significant association between venous thrombosis and increased mortality rates in patients undergoing cardiac surgery (6). However, nowadays, whether SGLT2 inhibition could reduce the risk of venous thrombosis is unclear.

In light of this, a Mendelian randomization (MR) study was required to explore whether SGLT2 inhibition was significantly linked to the risk of cardiac events-induced death via the mediation of venous thrombosis at a genetic level. Furthermore, we investigated the causal relationship between SGLT2 inhibitors and cardiac arrest as well as CHD.

MR represents a robust methodology that employs genetic variants linked to the exposure serving as instruments to explore the potential cause-effect connections between exposure and outcome (7). MR could emulate randomized controlled trials by allocating genetic variants randomly during conception, thereby reducing the likelihood of bias.

## Methods

### Study design

**Figure 1:**
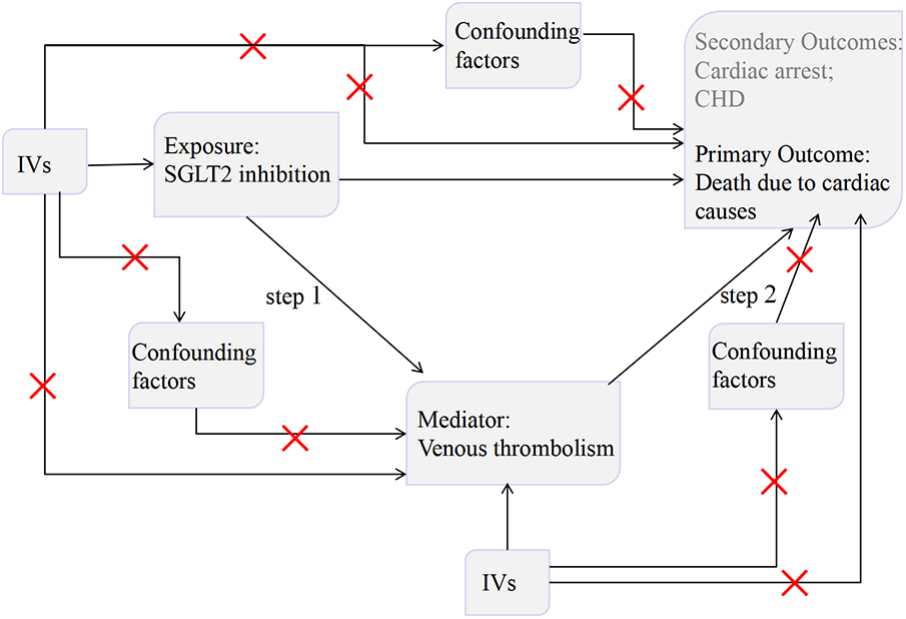
Schematic representation of the MR study.

The present study utilized an MR design (Figure 1). To establish the accuracy of potential cause-effect connections, three fundamental assumptions must be complied with in MR studies (7): genetic variants should (1) be powerfully linked to the exposure, (2) be independent of any potential confounding factors, (3) exert impact on outcome solely through exposure.

We first performed a two-sample MR to assess the relationship between SGLT2 inhibition on death due to cardiac causes. Then, we employed a two-step MR approach to ascertain the potential causal involvement of venous thrombolism in mediating the pathway between SGLT2 inhibition and death due to cardiac causes. Finally, we utilized two-sample MR to investigate the causal relationships between SGLT2 inhibition and the incidence of cardiac arrest and CHD.

### The selection of valid genetic variants for SGLT2 inhibition

The systematic identification of genetic variants acting as representatives for SGLT2 inhibition was conducted through a four-step process. (1) Choose genetic variants linked to SLC5A2 mRNA employing data from Genotype-Tissue Expression (GTEx) (8) and eQTLGen Consortium (9). (2) Evaluate the correlation between genetic variants selected with hemoglobin A1c (HbA1c) level, which serve as marker of the glucose-lowering effect resulting from SGLT2 inhibition and identify variants displaying regional-scale connection with HbA1c utilizing data deriving from a non-diabetic subset of independent individuals of European descent in the UK Biobank (*n* = 344,182) (*P* = 1 × 10^−4^). (3) Employ the genetic colocalization method to ascertain whether SLC5A2 and HbA1c share a common cause-effect variant. Colocalization, as a statistical genetic method, utilizes a Bayesian model to assess a posteriori probability of a variant having shared cause-effect on two traits: SLC5A2 expression and HbA1c level in circulation within the genomic locus of SLC5A2. The possibility of colocalization exceeding 70% between the gene expression of SLC5A2 and the levels of HbA1 was an indicative proof of colocalization, denoted as “colocalized”. Conversely, “not colocalized” was used to describe the remaining gene-disease associations. (4) Perform a standard clumping process (whereby variants with a correlation greater than 0.8 were eliminated as they exhibited excessively high correlations among one another). Then, the statistical power of each genetic variant selected was estimated using F statistics. Eventually, six single nucleotide polymorphisms (SNPs) forcefully linked to SGLT2 inhibition through HbA1c were identified as the instruments of the exposure (10) (Supplementary Table S1).

### Genetic instruments for venous thrombolism

Summary data for venous thrombolism was retrieved from a genome-wide association study (GWAS) (https://gwas.mrcieu.ac.uk/), (ebi-a-GCST90038607) containing 12,240 venous thrombolism cases and 472,358 controls of European descent with 9,587,836 SNPs. Genetic variants demonstrating significant associations with venous thrombolism (*P* < 5 × 10^−8^) were identified and subsequently considered as potential genetic predictors for venous thrombolism. We then performed a clumping procedure to exclude genetic variants that displayed significant intercorrelation (with a correlation coefficient <0.001) among themselves. Finally, thirteen SNPs connected with venous thrombolism were designated as genetic instruments of venous thrombolism. Then, we estimated the strength of thirteen variants using F statistics. An F-statistic threshold of less than 10 was employed to classify an instrumental variable (IV) as weak, and the weak IVs were excluded from our study. In addition, we applied the PhenoScanner web (http://www.phenoscanner.medschl.cam.ac.uk/) to analyze the selected genetic predictors and removed the SNPs related to potential confounding factors (Low-density lipoprotein, coronary artery disease) for cardiac events related to death. Finally, seven genetic variants vigorously connected with venous thrombolism were regarded as valid IVs (Supplementary Table S2).

### Study outcome

A GWAS data (ukb-d-I9_K_CARDIAC) was used as the summary association statistics of death due to cardiac causes, involving a European descent population consisting of 1,597 cases and 359,597 controls. Mortality resulting from cardiac disease is the primary outcome, with cardiac arrest and CHD investigated as secondary outcomes. Cardiac arrest data was sourced from the Finngen, incorporating 2,471 cases and 210,652 controls. The CHD data originated from the UK Biobank, incorporating 361,194 individuals of European descent.

## Statistical analyses

### MR analysis of SGLT2 inhibition on death due to cardiac causes

First, we assessed the impact of SGLT2 inhibition on death due to cardiac causes. We ran an MR study using five approaches: inverse variance weighted (IVW), MR-Egger, weighted median, simple mode, and weighted mode (11–13). The IVW approach is widely regarded as the most accurate method to assess causality, particularly in cases where there is no indication of directional pleiotropy in the findings (*P* for MR-Egger intercept *P* > 0.05). Due to its robustness, the IVW method is frequently employed as the chief analytical approach in the field of causal evaluation (14). The MR-Egger method possesses the capability to detect and account for directional pleiotropy. Yet, it demonstrated an inadequate statistical power (15). For the utilization of the weighted median approach to estimate cause-effect, a minimum criterion of considering 50% of SNPs was deemed reliable IVs (12). The simple mode represents a model-based evaluation method that provides robustness against pleiotropy (16). The weighted mode is susceptible to challenges in selecting an appropriate bandwidth for mode estimation (17). We verified the consistency of the causal associations using all five approaches collectively and established their statistical significance by employing a significance threshold of *P* < 0.05 (18, 19).

### Mediation MR analysis linking SGLT2 inhibition with death due to cardiac causes via venous thrombolism

In our study, the causal connection of SGLT2 inhibition on venous thrombolism was evaluated. The six SNPs imitating SGLT2 inhibition were regarded as the exposure, and the venous thrombolism was utilized as an outcome. We used five approaches to assess the cause-effect of SGLT2 inhibition on venous thrombolism. Among them, IVW was considered as our main approach (β1).

In addition, we evaluated the causal relationship between venous thrombolism on death due to cardiac causes. The seven variants robustly related to venous thrombolism were regarded as IVs for the exposure, and death due to the cardiac cause was selected as the outcome. We used five approaches to assess the effect of venous thrombolism on death because of cardiac causes. Amongst them, IVW was our main approach (β2).

Lastly, we used the product of coefficients method as a primary approach to assessing the indirect impact of SGLT2 inhibition on death due to cardiac cause via venous thrombolism (β1 × β2). Hence, the proportion of the impact mediated by venous thrombolism was calculated as the ratio between the mediated effect and the overall effect.

### Other MR assessment

To ensure the accuracy of our MR results, we conducted a comprehensive set of other MR examinations, consisting of sensitivity, heterogeneity, and pleiotropy assessment. These additional investigations were aimed at addressing possible biases arising from variations and polymorphisms among individual SNPs. To detect potential heterogeneity in MR analysis, we used Cochran's *Q* test, a recognized statistical method (20). Subsequently, we employed the “leave-one-out” approach for assessing the cause-effect genetically of exceptional SNPs and determining whether the non-inclusion of these SNPs had any impact on the MR results. This approach assisted us in evaluating the sensitivity of our findings to the presence or absence of specific SNPs in the analysis (21). To measure potential directional pleiotropy, we examined the intercept coefficient in the MR-Egger's regression analysis. A significant deviation of the intercept coefficient from zero (*p* < 0.05) would indicate the presence of directional pleiotropy, whereby the genetic variants have effects on the outcome that are independent of the exposure variable (11). Additionally, to assess for horizontal pleiotropy, and identify, and account for potential outliers, we employed the MR-PRESSO approach (19).

### Biological pathway evidence

For a more comprehensive understanding of the mechanisms underlying the association between SGLT2 inhibition and venous thrombosis, we carried out additional investigations by leveraging protein correlation data sourced from the STRING database. To construct a protein-protein interaction (PPI) network, we curated proteins from two distinct sources: (1) gene SLC5A2, which is associated with SGLT2, (2) 117 proteins associated with venous thrombolism from the Disgenet website, (3) first neighbors of SLC5A2 from 117 proteins. The protein network was constructed using the stringApp plugin within the Cytoscape platform.

## Result

### Effect of SGLT2 inhibition on death due to cardiac causes

**Figure 2:**
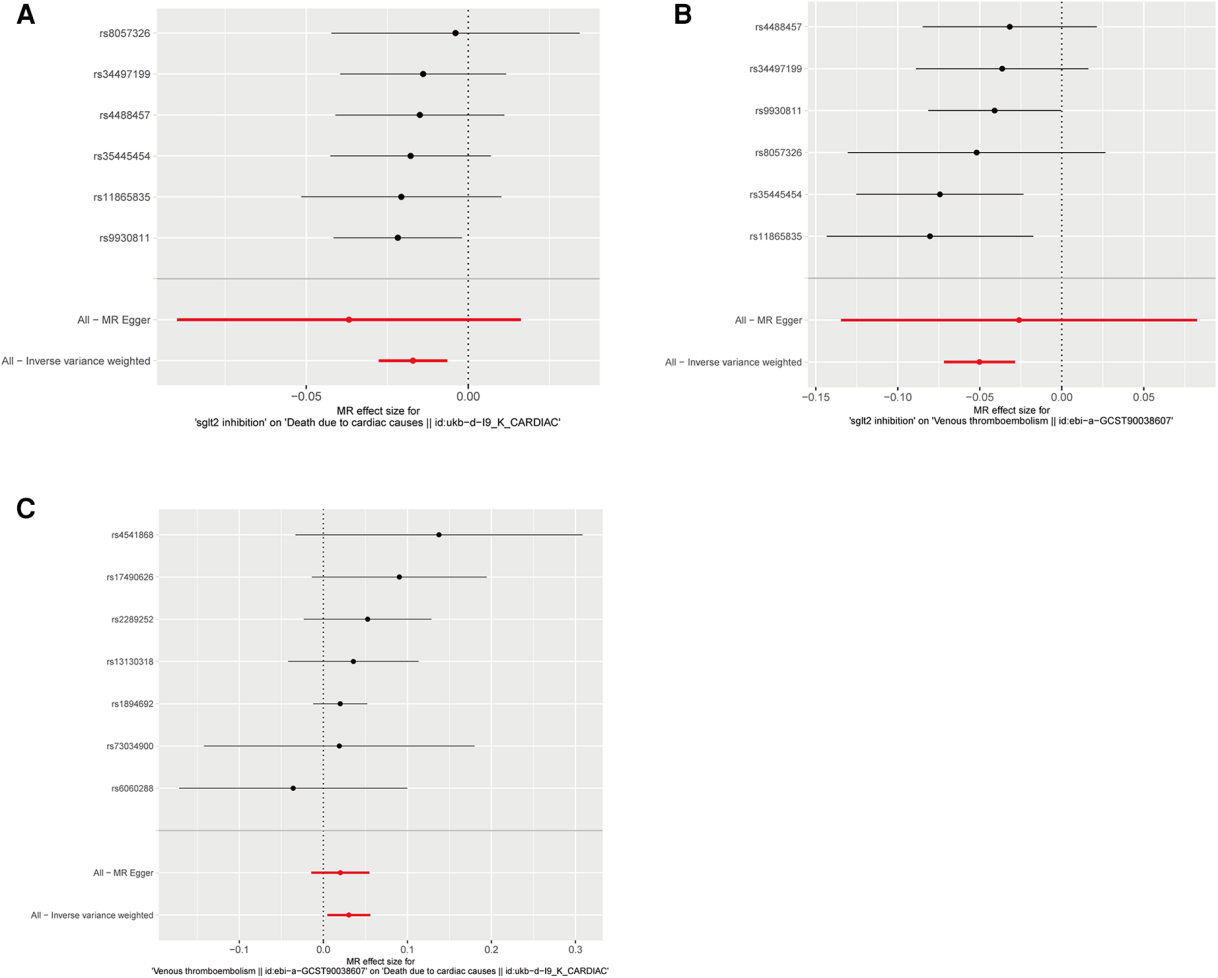
Forest plots of Mendelian randomization analyses in our study. (**A**) Forest plot of Mendelian randomization analyses of the causal effects of SGLT2 Inhibition on death due to cardiac causes; (**B**) Forest plot of Mendelian randomization analyses of the causal effects of SGLT2 inhibition on venous thrombolism; (**C**) Forest plot of venous thrombolism on the risk for death due to cardiac causes.

**Figure 3:**
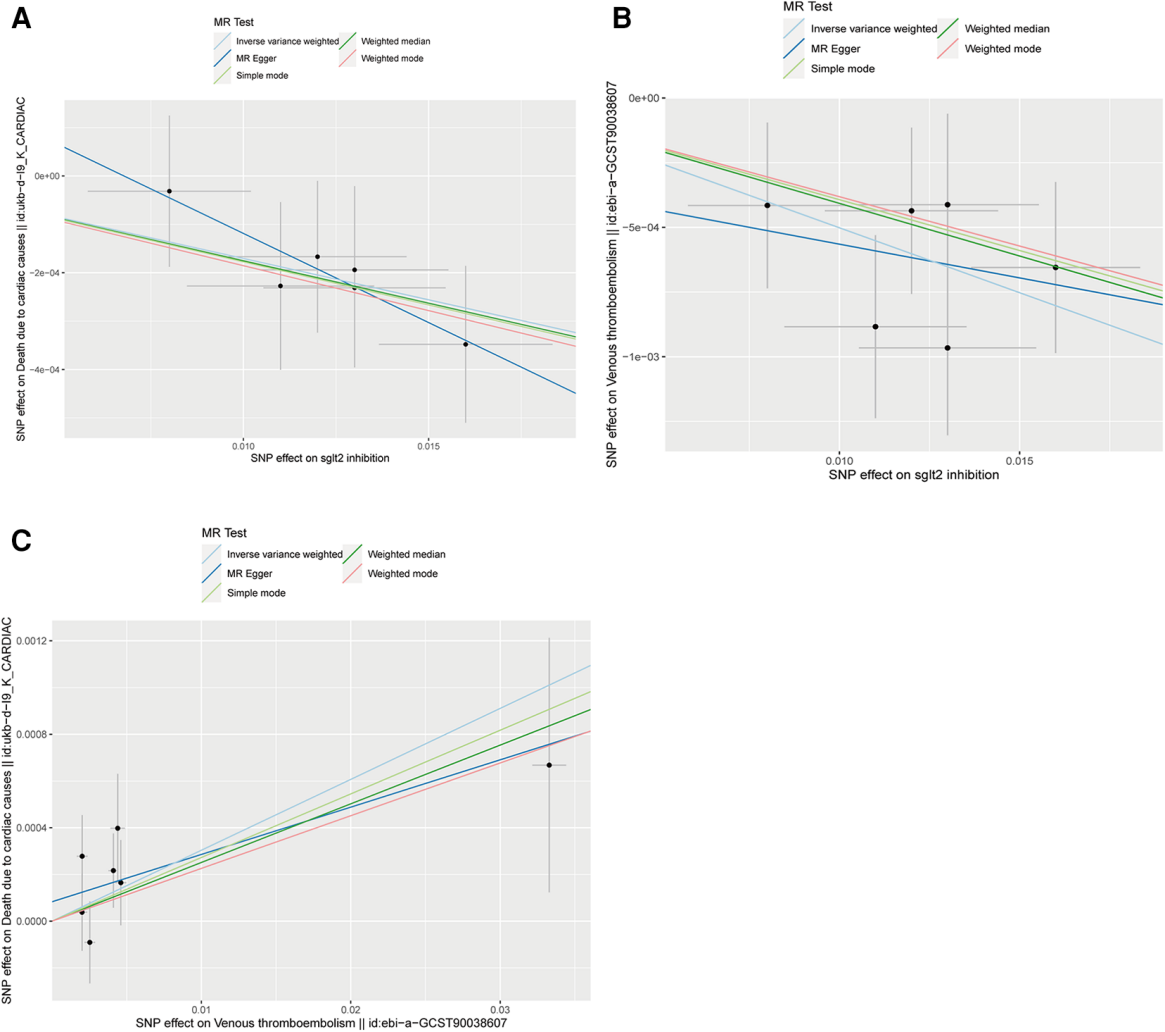
Scatter plots of Mendelian randomization analyses in our study. (**A**) Scatter plot of Mendelian randomization analyses of the causal effects of SGLT2 Inhibition on death due to cardiac causes; (**B**) Scatter plot of Mendelian randomization analyses of the causal effects of SGLT2 inhibition on venous thrombolism; (**C**) Scatter plot of venous thrombolism on the risk for death due to cardiac causes.

**Figure 4:**
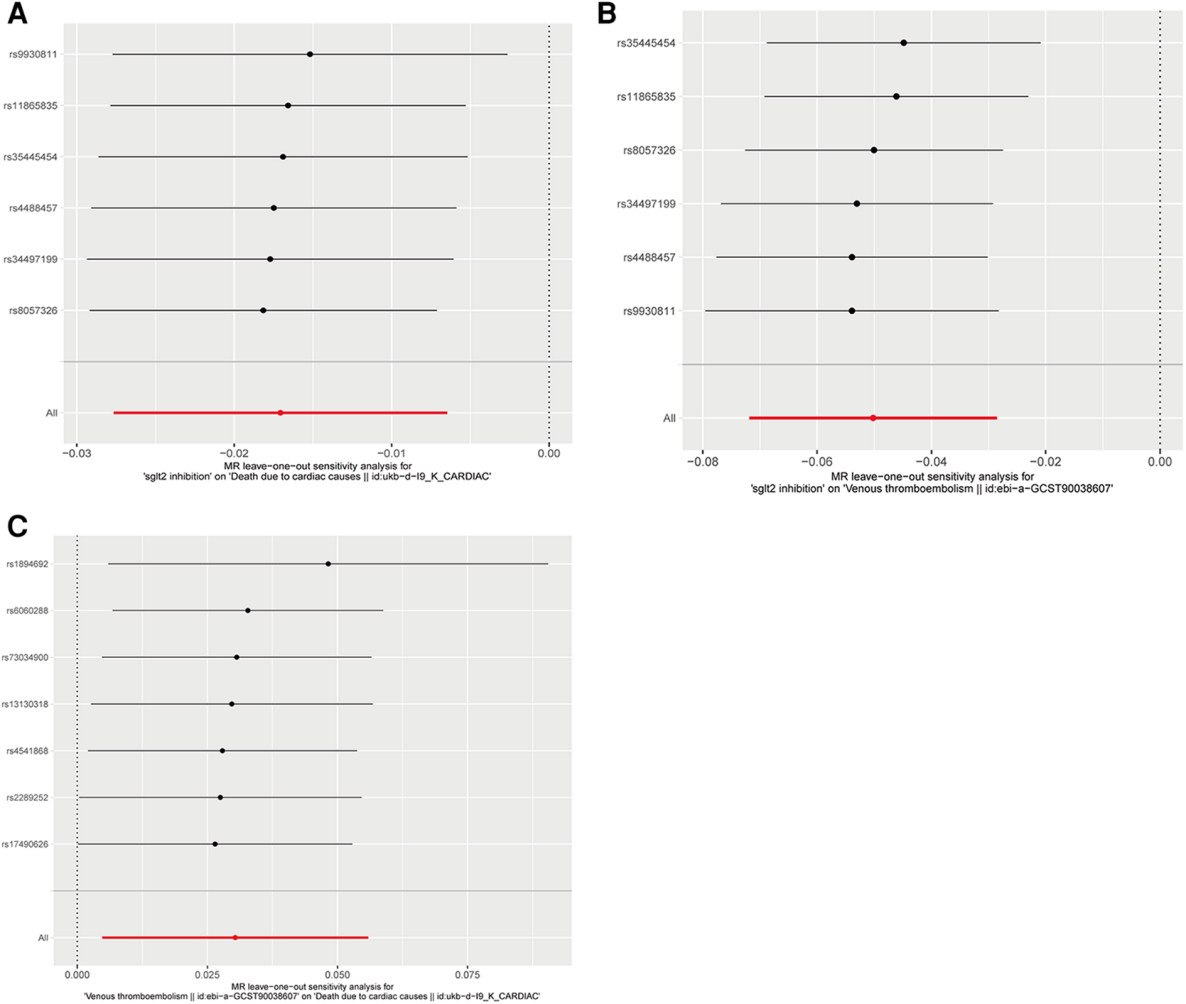
Leave-one-out plots of Mendelian randomization analyses in our study. (**A**) Leave-one-out plot of Mendelian randomization analyses of the causal effects of SGLT2 Inhibition on death due to cardiac causes; (**B**) Leave-one-out plot of Mendelian randomization analyses of the causal effects of SGLT2 inhibition on venous thrombolism; (**C**) Leave-one-out plot of venous thrombolism on the risk for death due to cardiac causes.

The IVW approach was employed as the chief analysis method in this study. SGLT2 inhibition was found to be connected with decreased hazard of mortality attributed to cardiac causes [OR = 0.983, (95% CI = 0.972, 0.993), *P* = 0.0016], and the weighted median approach yielded additional evidence to substantiate this finding [OR = 0.982, (95% CI = 0.969, 0.996), *P* = 0.0127]. Elaborate assessments can be found in Supplementary Table S3, accompanied by visual representations such as forest plots and scatter plots (Figures 2A, 3A). Moreover, the heterogeneity analysis utilizing Cochran's *Q* test for the IVW method demonstrated that the *Q* statistics and corresponding *p*-values did not exhibit statistical significance (*P* = 0.98), which suggested no proof of heterogeneity for the conclusion of SGLT2 inhibition on death due to cardiac causes. The MR-Egger regression analysis provided evidence that our findings remained unaffected by any potential pleiotropy (intercept *P* = 0.497), and the MR-PRESSO method indicated that our results were not affected by pleiotropy (Global Test *P* = 0.976). The leave-one-out plot, depicted in Figure 4A, illustrated that eliminating an individual SNP from the set of genetic variants had a minimal impact on the overall outcome.

### Mediation MR of SGLT2 inhibition, venous thrombolism, death due to cardiac causes

In this study, the cause-effect of SGLT2 inhibition on venous thrombolism was estimated. SGLT2 inhibition demonstrated a significant association with a decreased risk of venous thrombolism [OR =  0.951, (95% CI = 0.931, 0.972), *P* = 0.0000057]. Elaborate assessments can be found in Supplementary Table S4, accompanied by visual representations such as forest plots and scatter plots (Figures 2B, 3B). The heterogeneity analysis utilizing Cochran's *Q* test for the IVW method demonstrated that the *Q* statistics and corresponding *p*-values did not exhibit statistical significance (*P* = 0.750), which suggested no proof of heterogeneity for the conclusion of SGLT2 inhibition on venous thrombolism. The MR-Egger regression analysis provided evidence that our findings remained unaffected by any potential pleiotropy (intercept *P* = 0.680), and the MR-PRESSO method indicated that our results were not affected by pleiotropy (Global Test *P* = 0.798). The leave-one-out plot, depicted in Figure 4B, illustrated that eliminating an individual SNP from the set of genetic variants had a minimal impact on the overall outcome.

Then, we evaluated the effect of venous thrombolism on the risk of death due to cardiac causes. Venous thrombolism was linked to a higher mortality rate due to cardiac causes [OR =  1.031, (95% CI = 1.005, 1.057), *P* = 0.0199]. Elaborate assessments can be found in Supplementary Table S5, accompanied by visual representations such as forest plots and scatter plots (Figures 2C, 3C). The heterogeneity analysis utilizing Cochran's *Q* test for the IVW method demonstrated that the Q statistics and corresponding *p*-values did not exhibit statistical significance (*P* = 0.612), which suggested no proof of heterogeneity for the conclusion of venous thrombolism on death due to cardiac causes. The MR-Egger regression analysis provided evidence that our findings remained unaffected by any potential pleiotropy (intercept *P* = 0.432), and the MR-PRESSO method indicated that our results were not affected by pleiotropy (Global Test *P* = 0.626). The leave-one-out plot, depicted in Figure 4C, illustrated that eliminating an individual SNP from the set of genetic variants had a minimal impact on the overall outcome.

We detected a mediated impact of SGLT2 inhibition on death due to cardiac causes through venous thrombolism [β = −0.0015, (95% CI = −0.0032 −0.0002), *P* = 0.042], with a mediated proportion of 8.9% (95% CI = 1.2%, 18.7%) of the total effect (Table 1).

**Table 1:** The mediation effect of SGLT2 inhibition on death due to cardiac cause through venous thrombolism.

Mediator	Total effect	Direct effect A	Direct effect B	Mediation effect		Mediated proportion (%) (95% CI)
	β (95% CI)	β (95% CI)	β (95% CI)	β (95% CI)	*P*	
venous thrombolism	−0.0171 (−0.0277, −0.0065)	−0.0502 (−0.0719, −0.0285)	0.0304 (0.0048, 0.0559)	−0.0015 (−0.0032, −0.0002)	0.042	8.9 (1.2, 18.7)

“Total effect” indicates the effect of SGLT2 inhibition on death due to cardiac causes, “Direct effect A” indicates the effect of SGLT2 inhibition on venous thrombolism, “Direct effect B” indicates the effect of venous thrombolism on death due to cardiac causes and “mediation effect” indicates the effect of SGLT2 inhibition on death due to cardiac causes through venous thrombolism. Total effect, direct effect A and direct effect B were derived by IVW; mediation effect was derived by using the product of coefficients method. All statistical tests were two-sided. *P* < 0.05 was considered significant.

### PPI network

**Figure 5:**
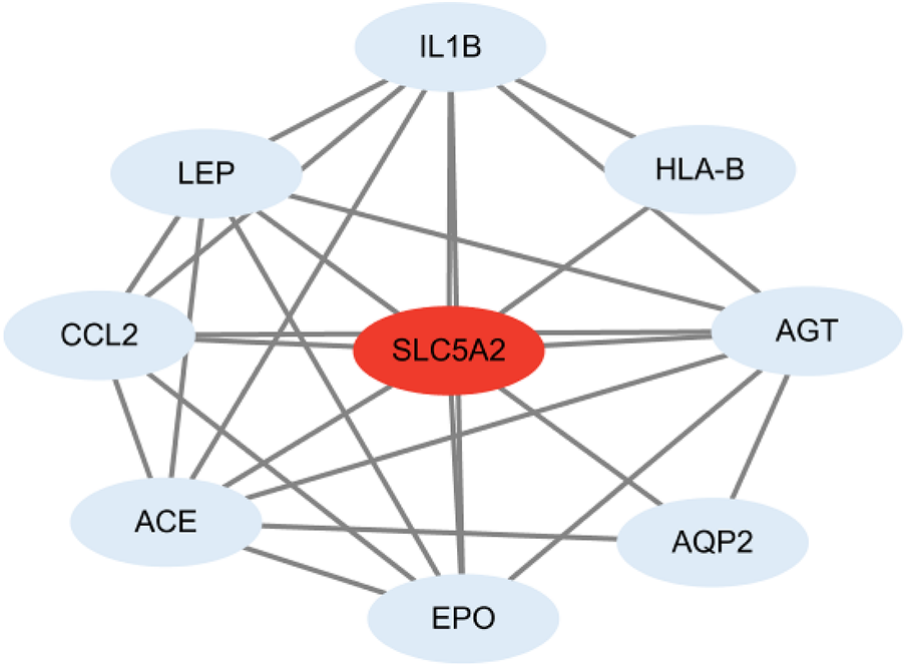
PPI network.

We performed protein-protein interaction analysis between SLC5A2 and the obtained proteins associated with venous thrombolism from the Disgenet website and identified eight proteins that primarily interact with SLC5A2, including interleukin 1 beta (IL1B), major histocompatibility complex, class I, B (HLA-B), angiotensinogen (AGT), aquaporin 2 (AQP2), erythropoietin (EPO), angiotensin I converting enzyme (ACE), C-C motif chemokine ligand 2 (CCL2), leptin (LEP) (Figure 5).

### Effect of SGLT2 inhibition on cardiac arrest

MR results demonstrated that SGLT2 inhibition was negatively associated with cardiac arrest [OR =  0.097, (95% CI = 0.013, 0.742), *P* = 0.025, IVW]. Elaborate assessments can be found in Supplementary Table S6, accompanied by visual representations such as forest plots and scatter plots (Supplementary Figures S1A,B). Moreover, the heterogeneity analysis utilizing Cochran's *Q* test for the IVW method demonstrated that the *Q* statistics and corresponding *p*-values did not exhibit statistical significance (*P* = 0.917), which suggested no proof of heterogeneity for the conclusion of SGLT2 inhibition on cardiac arrest. The MR-Egger regression analysis provided evidence that our findings remained unaffected by any potential pleiotropy (intercept *P* = 0.615), and the MR-PRESSO method indicated that our results were not affected by pleiotropy (Global Test *P* = 0.929). The leave-one-out plot, depicted in Supplementary Figure S1C, illustrated that eliminating an individual SNP from the set of genetic variants had a minimal impact on the overall outcome.

### Effect of SGLT2 inhibition on CHD

This study showed that SGLT2 inhibition was negatively linked to CHD [OR =  0.957, (95% CI = 0.932, 0.982), *P* = 0.0009, IVW]. Elaborate assessments can be found in Supplementary Table S7, accompanied by visual representations such as forest plots and scatter plots (Supplementary Figures S1D,E). Moreover, the heterogeneity analysis utilizing Cochran's *Q* test for the IVW method demonstrated that the *Q* statistics and corresponding *p*-values did not exhibit statistical significance (*P* = 0.650), which suggested no proof of heterogeneity for the conclusion of SGLT2 inhibition on CHD. The MR-Egger regression analysis provided evidence that our findings remained unaffected by any potential pleiotropy (intercept *P* = 0.763), and the MR-PRESSO method indicated that our results were not affected by pleiotropy (Global Test *P* = 0.682). The leave-one-out plot, depicted in Supplementary Figure S1F, illustrated that eliminating an individual SNP from the set of genetic variants had a minimal impact on the overall outcome.

## Discussion

**Figure 6:**
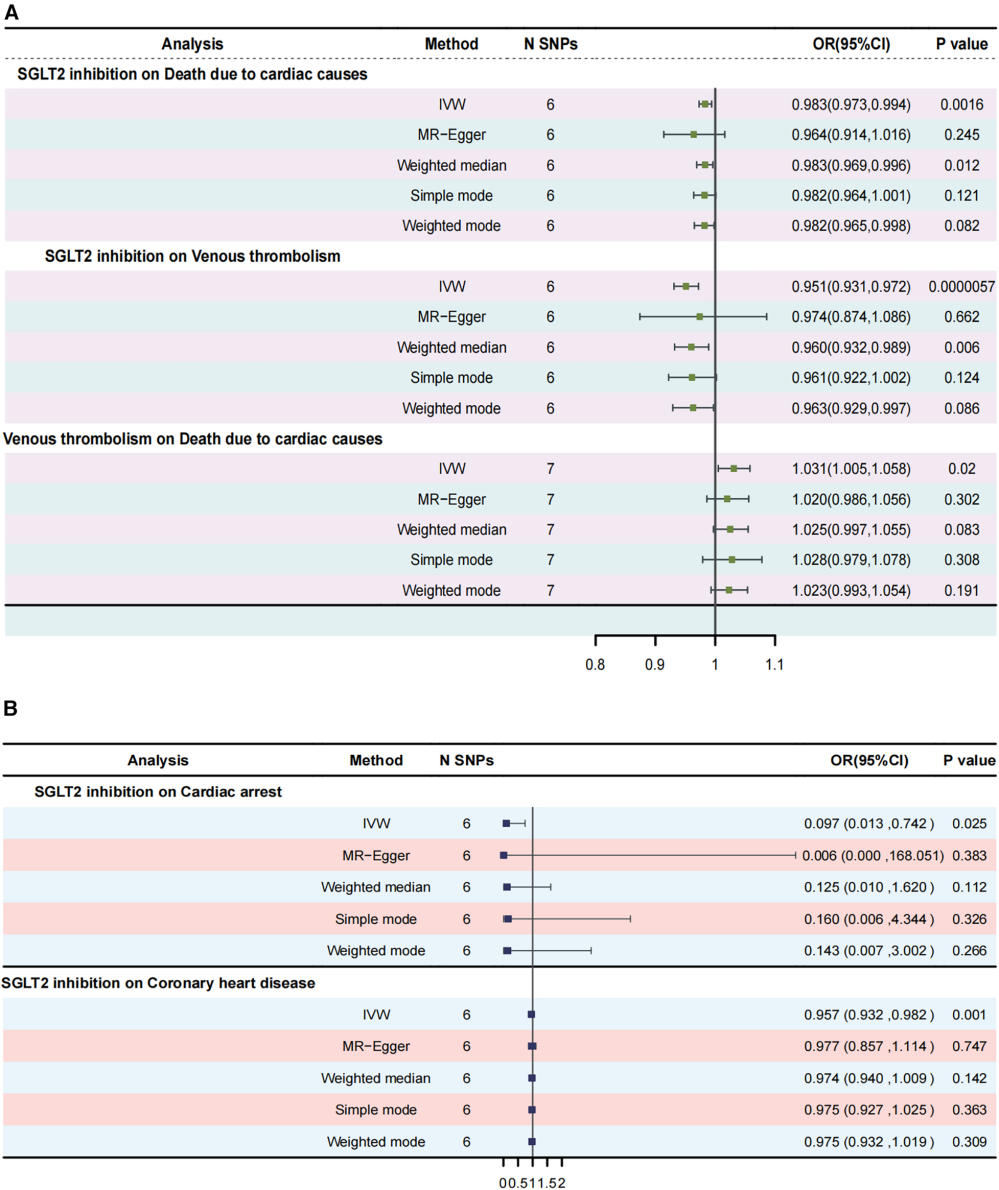
Summary of Mendelian randomization analyses in our study. (**A**) Mendelian randomization analyses among SGLT2 inhibition, venous thrombolism as well as death due to cardiac causes; (**B**) Mendelian randomization analyses for SGLT2 inhibition on cardiac arrest and CHD. CHD, coronary heart disease.

In this study, we investigated and established the causal effects of SGLT2 inhibition on both venous thrombolism and death due to cardiac causes. Additionally, we estimated the causal impact of venous thrombolism on death due to cardiac causes (Figure 6A). Moreover, the mediation MR study demonstrated the potential causal influence of SGLT2 inhibition on death due to cardiac causes via venous thrombolism. We also suggested that venous thrombolism was genetically linked to a raised hazard of mortality due to cardiac causes. Furthermore, our findings also demonstrate a negative correlation between SGLT2 inhibition and cardiac arrest as well as CHD (Figure 6B).

A meta-study has reported that among individuals diagnosed with heart failure characterized by reduced ejection fraction, the associations remained consistent irrespective of their diabetic condition, SGLT2 inhibition could reduce the combined risk of cardiovascular death or hospitalisation (22). Another system review and meta-analysis demonstrated that SGLT2 inhibition effectively mitigated the hazard of cardiac mortality, heart failure, and other cardiac events (23). A clinical study showed SGLT2 inhibition was also related to a significantly lower risk of cardiac arrest and CHD (24, 25). In our study, we have shown the causal effects of SGLT2 inhibition on death due to cardiac causes, cardiac arrest as well as CHD. However, limited research has been conducted to investigate the underlying mechanisms responsible for the impact of SGLT2 inhibition on death attributed to cardiac causes.

Previous studies have shown that the amelioration of cardiovascular outcomes associated with empagliflozin, an SGLT2 inhibitor, is not solely reliant on glycemic control (26). This implies the existence of alternative mechanisms and pathways through which the effects are mediated.

Venous thromboembolism is the third main contributor to cardiovascular death in the world (27). In this MR analysis, it has been demonstrated that venous thrombolism is a genetic risk for cardiac events related to death. The potential mechanism is related to vascular endothelial injury, hypercoagulability, and abnormal bloodstream dynamics. Vascular endothelial injury is the important initiation of venous thrombosis. Blood flow disturbances may encompass hypercoagulability of the blood as well as abnormal coagulation states within the vessel wall. Heart diseases often lead to vascular endothelial damage (28), platelet activation, a hypercoagulable state (29), and blood stasis (30).

In addition, up to now, the existing research on the effect of SGLT2 inhibition on venous thromboembolism is in conflict. A pilot study showed that SGLT2 Inhibitors reduced platelet activation and thrombus formation (31). However, another study indicated that the administration of SGLT2 inhibitors has been linked to elevated levels of hematocrit, leading to augmented blood viscosity (32), and, consequently, elevating the potential risk of thromboembolic events (33). Yet, according to a cohort study, the use of SGLT2-targeted medicine in individuals diagnosed with type 2 diabetes was not found to be correlated with an increased incidence rate of venous thromboembolism (34). Additionally, a systematic review and meta-analysis also demonstrated no influence of SGLT2 inhibitors on the incidence of venous thromboembolism in diabetic patients (35). Conflict results may be related to the limitations of different studies. Most studies did not fully adjust for confounding factors and the definition of venous thromboembolism varied across trials. Therefore, we used MR analysis to explore the causal relationship between SGLT2 inhibition and venous thrombolism. We found that SGLT2 inhibition could reduce the risk of venous thrombolism. Further, our mediation MR analysis suggested venous thrombolism mediated the effects of SGLT2 inhibition on death due to cardiac causes with an indirect component of 8.9%. However, the potential mechanism of SGLT2 inhibitors on venous thrombolism remains unclear. We obtained proteins related to venous thrombolism from the Digenet website and selected the proteins which were the first neighbors of SLC5A2. As Figure 5, IL1B, HLA-B, AGT, AQP2, EPO, ACE, CCL2, LEP were related to SLC5A2. IL1B and CCL2 are linked to inflammation. AGT, EPO, LEP, and ACE participate in regulating platelet activity and coagulation process (36, 37). Inflammation could promote platelet hyperreactivity (38) and upregulate pathological thrombosis (39). Therefore, SGLT2 inhibition may inhibit vein thrombolism by regulating inflammation, platelet activation, and the coagulation process.

However, in our study, there is an apparent limitation. The odds ratios of our MR results are quite mild. The potential reasons are following. Firstly, the number of instrumental SNPs used is associated with the OR value to some extent; however, in this research, only six SNPs were employed as proxies for the exposure. Secondly, the OR value is also related to the proportion of cases and controls within outcome data, and in our study, the numbers of cases in the outcome datasets are relatively small. This could potentially lead to mild OR estimates in our investigation. Therefore, the putative link between venous thrombolism-mediated effects of SGLT2 inhibitors and their contribution to cardiovascular mortality requires additional empirical validation through dedicated experiments.

## Conclusion

Genetically, SGLT2 inhibition could reduce the mortality rates due to cardiac events via venous thrombolism. The comprehensive mechanism may relate to the effects of SGLT2 inhibition on inflammation, platelet activation, and the coagulation process. Addtionally, SGLT2 inhibition could reduce the risks of cardiac arrest and CHD.

## Data Availability

The datasets presented in this study can be found in online repositories. The names of the repository/repositories and accession number(s) can be found in the article/**Supplementary Material**.

## Supplementary material

Supplementary Figure S1 MR analyses plots for SGLT2 inhibition on cardiac arrest and CHD. (A) Forest plot of Mendelian randomization analyses of the causal effects of SGLT2 Inhibition on cardiac arrest; (B) Scatter plot of Mendelian randomization analyses of the causal effects of SGLT2 inhibition on cardiac arrest; (C) Leave-one-out plot of SGLT2 Inhibition on cardiac arrest; (D) Forest plot of Mendelian randomization analyses of the causal effects of SGLT2 Inhibition on CHD; (E) Scatter plot of Mendelian randomization analyses of the causal effects of SGLT2 inhibition on CHD; (F) Leave-one-out plot of SGLT2 Inhibition on CHD. CHD, coronary heart disease.
