# Supplementary material for: SGLT2 inhibition, venous thrombolism, and death due to cardiac causes: a mediation Mendelian randomization study
Source: Front Cardiovasc Med. 2024 May 13;11:1339094. doi: 10.3389/fcvm.2024.1339094 (PMC11128626; doi:10.3389/fcvm.2024.1339094)
Supplement: Supplementary file 1 [file Table1.docx]

**Supplementary Table 1: genetic variants selected as genetic predictors of SGLT2 inhibition**

| SNP | effect_allele | other_allele | eaf | beta | pval | F value |
| --- | --- | --- | --- | --- | --- | --- |
| rs4488457 | G | T | 0.712 | -0.013 | 2.90E-07 | 26.315 |
| rs8057326 | C | T | 0.523 | -0.008 | 2.80E-04 | 13.200 |
| rs11865835 | C | T | 0.284 | -0.011 | 1.34E-05 | 19.010 |
| rs9930811 | G | A | 0.365 | -0.016 | 8.69E-12 | 24.912 |
| rs34497199 | T | C | 0.475 | -0.012 | 5.98E-07 | 24.912 |
| rs35445454 | T | C | 0.344 | -0.013 | 1.24E-07 | 28.374 |

**Supplementary Table 2: genetic variants selected as genetic predictors of venous thrombolism**

| SNP | effect_allele | other_allele | eaf | beta | pval |
| --- | --- | --- | --- | --- | --- |
| rs1894692 | A | G | 0.979028 | -0.0332812 | 2.40E-191 |
| rs13130318 | G | T | 0.239859 | 0.00460632 | 2.30E-35 |
| rs2289252 | T | C | 0.395488 | 0.00410789 | 2.10E-36 |
| rs4541868 | A | C | 0.281626 | -0.00201957 | 9.00E-09 |
| rs17490626 | C | G | 0.123796 | -0.00439766 | 1.90E-19 |
| rs73034900 | C | T | 0.341638 | 0.0020107 | 3.60E-09 |
| rs6060288 | A | G | 0.286071 | 0.00253007 | 1.10E-12 |

**Supplementary Table 3: MR estimates of the effect of SGLT2 inhibition on death due to cardiac causes**

| Method | OR (95% CI) | P | Q statistic | P-heterogeneity | Egger intercept | P-intercept |
| --- | --- | --- | --- | --- | --- | --- |
| IVW | 0.983(0.973,0.994) | 0.0016 | 0.805 | 0.977 |  |  |
| MR-Egger | 0.964(0.914,1.016) | 0.245 | 0.249 | 0.993 | 0.0002 | 0.497 |
| Weighted median | 0.983(0.969,0.996) | 0.012 |  |  |  |  |
| Simple mode | 0.982(0.964,1.001) | 0.121 |  |  |  |  |
| Weighted mode | 0.982(0.965,0.998) | 0.082 |  |  |  |  |

IVW, inverse–variance weighted; P-heterogeneity, P value for heterogeneity test; P-intercept, P-value for the intercept of MR-Egger regression. Odds ratio (OR), 95% confidence interval (CI), and P values were calculated for the respective method of MR analysis. The heterogeneity test in the IVW methods was performed using Cochran’s Q statistic. P < 0.05 was considered significant.

**Supplementary Table 4: MR estimates of the effect of the effect of SGLT2 inhibition on venous thrombolism**

| Method | OR (95% CI) | P | Q statistic | P-heterogeneity | Egger intercept | P-intercept |
| --- | --- | --- | --- | --- | --- | --- |
| IVW | 0.951(0.931,0.972) | 0.0000057 | 2.678 | 0.750 |  |  |
| MR-Egger | 0.974(0.874,1.086) | 0.662 | 2.480 | 0.648 | -0.0003 | 0.680 |
| Weighted median | 0.960(0.932,0.989) | 0.006 |  |  |  |  |
| Simple mode | 0.961(0.922,1.002) | 0.124 |  |  |  |  |
| Weighted mode | 0.963(0.929,0.997) | 0.086 |  |  |  |  |

IVW, inverse–variance weighted; P-heterogeneity, P value for heterogeneity test; P-intercept, P value for the intercept of MR-Egger regression. Odds ratio (OR), 95% confidence interval (CI), and P values were calculated for the respective method of MR analysis. The heterogeneity test in the IVW methods was performed using Cochran’s Q statistic. P < 0.05 was considered significant.

**Supplementary Table 5: MR estimates of the effect of the effect of venous thrombolism on death due to cardiac causes**

| Method | OR (95% CI) | P | Q statistic | P-heterogeneity | Egger intercept | P-intercept |
| --- | --- | --- | --- | --- | --- | --- |
| IVW | 1.031(1.005,1.058) | 0.020 | 4.480 | 0.612 |  |  |
| MR-Egger | 1.020(0.986,1.056) | 0.302 | 3.750 | 0.586 | 8.301638e-05 | 0.432 |
| Weighted median | 1.025(0.997,1.055) | 0.083 |  |  |  |  |
| Simple mode | 1.028(0.979,1.078) | 0.308 |  |  |  |  |
| Weighted mode | 1.023(0.993,1.054) | 0.191 |  |  |  |  |

IVW, inverse–variance weighted; P-heterogeneity, P value for heterogeneity test; P-intercept, P value for the intercept of MR-Egger regression. Odds ratio (OR), 95% confidence interval (CI), and P values were calculated for the respective method of MR analysis. The heterogeneity test in the IVW methods was performed using Cochran’s Q statistic. P < 0.05 was considered significant.

**Supplementary Table 6: MR estimates of the effect of SGLT2 inhibition on cardiac arrest**

| Method | OR (95% CI) | P | Q statistic | P-heterogeneity | Egger intercept | P-intercept |
| --- | --- | --- | --- | --- | --- | --- |
| IVW | 0.097 (0.013 ,0.742 ) | 0.025 | 1.464 | 0.917 |  |  |
| MR-Egger | 0.006 (0.000 ,168.051 ) | 0.383 | 1.168 | 0.883 | 0.035 | 0.615 |
| Weighted median | 0.125 (0.010 ,1.620 ) | 0.112 |  |  |  |  |
| Simple mode | 0.160 (0.006 ,4.344 ) | 0.326 |  |  |  |  |
| Weighted mode | 0.143 (0.007 ,3.002 ) | 0.266 |  |  |  |  |

IVW, inverse–variance weighted; P-heterogeneity, P value for heterogeneity test; P-intercept, P-value for the intercept of MR-Egger regression. Odds ratio (OR), 95% confidence interval (CI), and P values were calculated for the respective method of MR analysis. The heterogeneity test in the IVW methods was performed using Cochran’s Q statistic. P < 0.05 was considered significant.

**Supplementary Table 7: MR estimates of the effect of SGLT2 inhibition on CHD**

| Method | OR (95% CI) | P | Q statistic | P-heterogeneity | Egger intercept | P-intercept |
| --- | --- | --- | --- | --- | --- | --- |
| IVW | 0.957 (0.932 ,0.982 ) | 0.001 | 3.328 | 0.650 |  |  |
| MR-Egger | 0.977 (0.857 ,1.114 ) | 0.747 | 3.223 | 0.521 | -0.0003 | 0.763 |
| Weighted median | 0.974 (0.940 ,1.009 ) | 0.142 |  |  |  |  |
| Simple mode | 0.975 (0.927 ,1.025 ) | 0.363 |  |  |  |  |
| Weighted mode | 0.975 (0.932 ,1.019 ) | 0.309 |  |  |  |  |

CHD, coronary heart disease; IVW, inverse–variance weighted; P-heterogeneity, P value for heterogeneity test; P-intercept, P-value for the intercept of MR-Egger regression. Odds ratio (OR), 95% confidence interval (CI), and P values were calculated for the respective method of MR analysis. The heterogeneity test in the IVW methods was performed using Cochran’s Q statistic. P < 0.05 was considered significant.
