# Supplementary figures and images for: SGLT2 inhibition, venous thrombolism, and death due to cardiac causes: a mediation Mendelian randomization study
Source: Front Cardiovasc Med. 2024 May 13;11:1339094. doi: 10.3389/fcvm.2024.1339094 (PMC11128626; doi:10.3389/fcvm.2024.1339094)

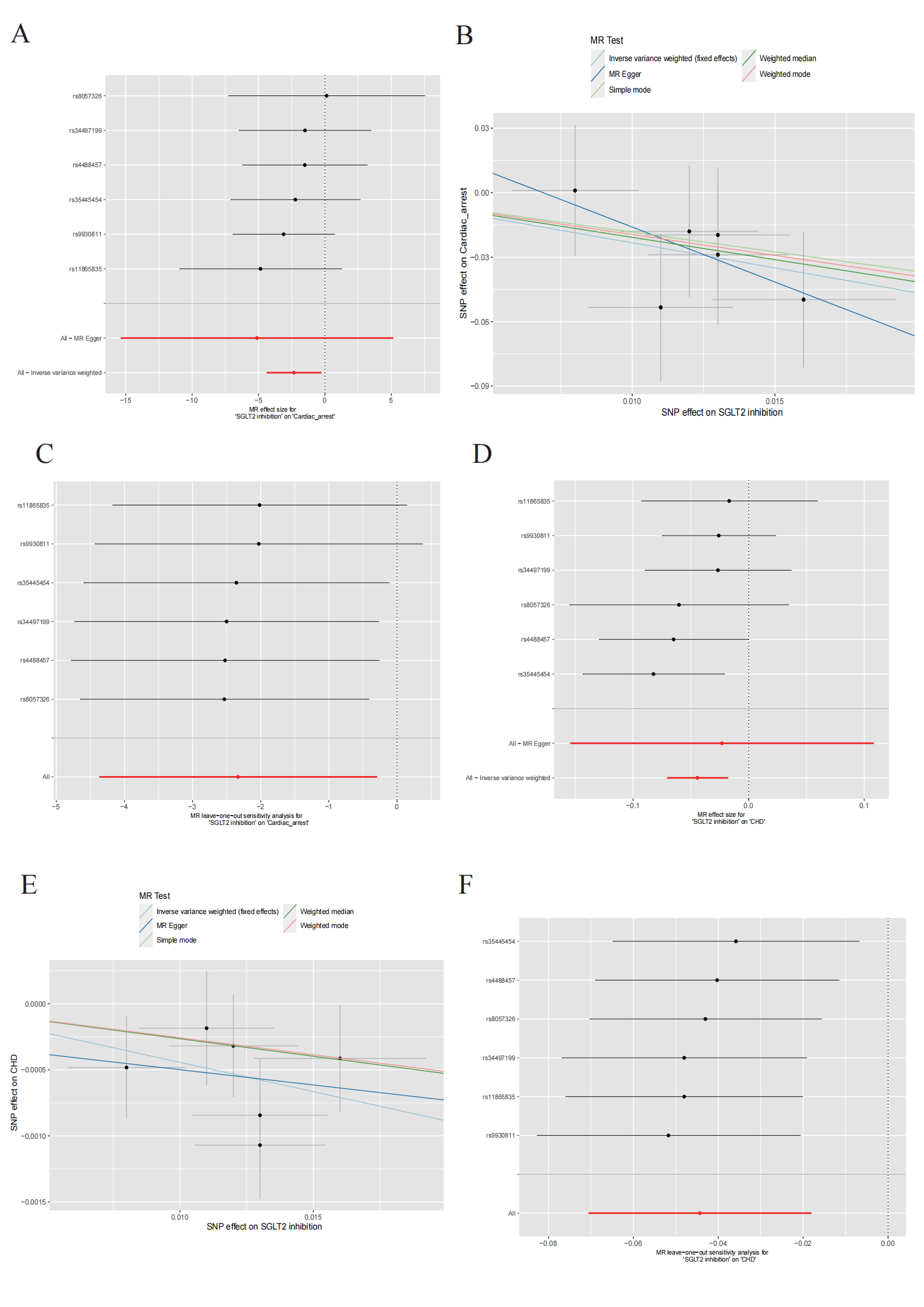

Supplement: Supplementary Figure S1 — MR analyses plots for SGLT2 inhibition on cardiac arrest and CHD. (A) Forest plot of Mendelian randomization analyses of the causal effects of SGLT2 Inhibition on cardiac arrest; (B) Scatter plot of Mendelian randomization analyses of the causal effects of SGLT2 inhibition on cardiac arrest; (C) Leave-one-out plot of SGLT2 Inhibition on cardiac arrest; (D) Forest plot of Mendelian randomization analyses of the causal effects of SGLT2 Inhibition on CHD; (E) Scatter plot of Mendelian randomization analyses of the causal effects of SGLT2 inhibition on CHD; (F) Leave-one-out plot of SGLT2 Inhibition on CHD. CHD, coronary heart disease. [file Image1.tif]
